# The influence of alkalosis on repeated high-intensity exercise performance and acid–base balance recovery in acute moderate hypoxic conditions

**DOI:** 10.1007/s00421-018-3975-z

**Published:** 2018-09-08

**Authors:** Lewis Anthony Gough, Danny Brown, Sanjoy K. Deb, S. Andy Sparks, Lars R. McNaughton

**Affiliations:** 1 Sport and Physical Activity Department, Faculty of Health and Life Sciences, Birmingham City University, Birmingham, B13 3TN UK; 2 Sports Nutrition and Performance Group, Department of Sport and Physical Activity, Edge Hill University, Ormskirk, Lancashire, L39 4QP UK; 3 Department of Sport and Movement Studies, Faculty of Health Science, University of Johannesburg, Johannesburg, South Africa

**Keywords:** Buffers, Alkalosis, Individual pursuit, Personalised nutrition, Track cycling

## Abstract

**Purpose:**

Exacerbated hydrogen cation (H^+^) production is suggested to be a key determinant of fatigue in acute hypoxic conditions. This study, therefore, investigated the effects of NaHCO_3_ ingestion on repeated 4 km TT cycling performance and post-exercise acid–base balance recovery in acute moderate hypoxic conditions.

**Methods:**

Ten male trained cyclists completed four repeats of 2 × 4 km cycling time trials (TT_1_ and TT_2_) with 40 min passive recovery, each on different days. Each TT series was preceded by supplementation of one of the 0.2 g kg^−1^ BM NaHCO_3_ (SBC2), 0.3 g kg^−1^ BM NaHCO_3_ (SBC3), or a taste-matched placebo (0.07 g kg^−1^ BM sodium chloride; PLA), administered in a randomized order. Supplements were administered at a pre-determined individual time to peak capillary blood bicarbonate concentration ([HCO_3_^−^]). Each TT series was also completed in a normobaric hypoxic chamber set at 14.5% FiO_2_ (~ 3000 m).

**Results:**

Performance was improved following SBC3 in both TT_1_ (400.2 ± 24.1 vs. 405.9 ± 26.0 s; *p* = 0.03) and TT_2_ (407.2 ± 29.2 vs. 413.2 ± 30.8 s; *p* = 0.01) compared to PLA, displaying a very likely benefit in each bout. Compared to SBC2, a likely and possible benefit was also observed following SBC3 in TT_1_ (402.3 ± 26.5 s; *p* = 0.15) and TT_2_ (410.3 ± 30.8 s; *p* = 0.44), respectively. One participant displayed an ergolytic effect following SBC3, likely because of severe gastrointestinal discomfort, as SBC2 still provided ergogenic effects.

**Conclusion:**

NaHCO_3_ ingestion improves repeated exercise performance in acute hypoxic conditions, although the optimal dose is likely to be 0.3 g kg^−1^ BM.

## Introduction

Repeated bouts of high-intensity exercise are a frequent feature of training and competition in athletes (Monedero and Donne 2000; Barnett 2006). The recovery between these exercise bouts is an essential component for determining the effectiveness of the subsequent bout. Enhanced recovery can allow athletes to tolerate greater training loads in the subsequent bout, potentially enhancing the post-training adaptation as a result (Barnett 2006). Whereas, in competition, enhancing recovery is an important component to sustain performance within the subsequent bout. This is applicable to sports such as track cycling, swimming, or a rowing regatta series which involve heats, semi-finals, and finals within a short amount of time (Al-Nawaiseh et al. 2016; Monedero and Donne 2000). Specifically, the gap between the men’s team pursuit first round and the final at the Rio 2016 Olympics was separated by just 60 min. Considering that most national and Olympic records are achieved within the preliminary rounds of these events (Al-Nawaiseh et al. 2016), this suggests that full recovery is not always possible during these time frames or that current recovery practices are not optimal. Therefore, interventions to improve recovery and sustain subsequent exercise performance are important.

A major factor that may hamper post-training recovery and the subsequent bout of exercise is the metabolic disturbance that occurs following an initial high-intensity exercise bout (Barnett 2006). Ward et al. (2016) reported that the decline in pH and HCO_3_^−^ following a 4 km cycling time trial (TT; team pursuit distance) was substantial, and reflective of metabolic acidosis (pH 7.16 ± 0.08, HCO_3_^−^ 11.9 ± 2.3 mmol l^−1^), which was measured using capillary blood samples. Full recovery of such disturbances is likely to take over 75 min, as Callahan et al. (2017) reported HCO_3_^−^ was 6.2 mmol l^−l^ below baseline at this point (19.5 ± 1.4 vs. 25.7 ± 1.0 mmol l^−1^). If only 60 min is available for recovery, therefore, such as that during track cycling events, an existing acid–base balance perturbation will be evident. Although contentious (Westerblad 2016), critical rises in hydrogen cation (H^+^) accumulation are linked to a reduction in both the release and uptake of calcium ions (Ca^2+^) from the sarcoplasmic reticulum (Allen et al. 2008), disruption of key enzymes of the glycolytic pathway (Hollidge-Horvat et al. 1999), and a reduction in muscle excitability and action potentials by reducing the strong ion difference (SID) (Cairns and Lindinger 2008). In turn, this may hamper subsequent performance by reducing the capability for muscle force production (Cairns 2006; Fitts 2016). It is intuitive to suggest, therefore, that interventions to accelerate post-exercise recovery of acid–base balance could be beneficial for a subsequent bout of exercise.

The ingestion of sodium bicarbonate (NaHCO_3_) has been shown to accelerate post-exercise acid–base balance recovery and subsequent exercise performance (Pruscino et al. 2008; Zabala et al. 2008, 2011; Gough et al. 2017a). Pruscino et al. (2008) reported a ‘trivial’ to ‘moderate’ benefit in the second bout of a 2 × 200 m freestyle swim (interspersed with a 30 min recovery) following pre-exercise ingestion of NaHCO_3_. It was then later reported that NaHCO_3_ ingestion 30 min into a 90 min post-exercise recovery significantly improved subsequent cycling capacity at 100% peak mean minute power by 16.6% (Gough et al. 2017a). Both studies reported that capillary blood pH and HCO_3_^−^ recovery were accelerated and above baseline at the end of the recovery period, whereas the placebo condition failed to fully recover. This time frame between bouts might explain why ergogenic effects were observed, as this allowed pH and HCO_3_^−^ to recover sufficiently. Conversely, no effect of NaHCO_3_ ingestion has been reported on three repeated Wingate tests separated shorter recovery time frames of between 15 and 30 min (Zabala et al. 2008, 2011), or during three repeated high-intensity swimming bouts separated by 20 min (Pierce et al. 1992). Zabala et al. (2008) showed that neither pH, nor HCO_3_^−^ recovered back to baseline levels between the three Wingate tests, suggesting that the recovery of pH and HCO_3_^−^ to this level may be important to produce ergogenic effects in the subsequent exercise bouts.

Despite the promising effects of NaHCO_3_ ingestion to improve repeated bouts of exercise in normoxia, this strategy has yet to be applied to acute hypoxia. Nonetheless, Robergs et al. (2005) reported the use of NaHCO_3_ combined with sodium citrate lead to post-exercise recovery of pH and HCO_3_^−^ to baseline in approximately 50 min at 1570 m terrestrial altitude, whereas the placebo condition failed to recover to baseline within the 80 min sampling period. Robergs et al. (2005) featured no subsequent bout of exercise, however, and it is also unclear if the participant cohort completed an acclimatisation period in the terrestrial altitude location. This enhanced post-exercise recovery displayed by Robergs et al. (2005) is potentially important, nonetheless, as athletes may complete multiple bouts of high-intensity exercise to maximise the adaptation from hypoxic training schedules, therefore, highlighting the need for optimal recovery. The use of pre-exercise NaHCO_3_ ingestion may, in turn, result in a blunting of the initial stress of acid–base balance during an initial bout of exercise, but also improve recovery, leading to improved subsequent exercise performance. This may have a cumulative effect in sustaining training volume and intensity during hypoxic training schedules, particularly considering chronic NaHCO_3_ ingestion has been shown to be effective at improving performance following training schedules at sea level (Egger et al. 2014; Durkalec-Michalski et al. 2018). The aim of this study, therefore, was to investigate the effects of both 0.2 g kg^−1^ BM and 0.3 g kg^−1^ BM NaHCO_3_ on repeated bouts of 4 km TT cycling performance in acute moderate hypoxic conditions. The hypothesis of this study was that both doses of NaHCO_3_ would improve both bouts of exercise compared to the placebo; however, 0.3 g kg^−1^ BM NaHCO_3_ would improve performance to the greatest extent.

## Methods

### Participants and compliance with ethical standards

Ten trained male cyclists (age 27 ± 8 years, body mass 82 ± 9 kg, hypoxic maximal rate of oxygen uptake (*V*O_2max_) 48.5 ± 5.6 ml kg min^−1^, and hypoxic peak power output 331 ± 40 W) volunteered for this study. All participants’ training load was reflective of a ‘trained’ cyclist (De Pauw et al. 2013). Ethical approval was granted from the Universities Research Ethics Committee (URESC16-LG01; Edge Hill University), and all participants provided written informed consent.

### Experimental overview

Using methods previously described (Gough et al. 2017b), an initial maximal rate of oxygen uptake (*V*O_2max_) test was conducted in a normobaric hypoxic chamber set at a fraction of inspired oxygen (FiO_2_) of 14.5% (~ 3000 m). Participants then visited the laboratory on a further six separate occasions in a block randomized, crossover, and double-blind designed study (2 × identification of peak blood HCO_3_^−^, and 4 × cycling TTs). Individual time to peak HCO_3_^−^ was determined prior to the cycling time trials, using a previously described method (Gough et al. 2017c). This entailed participants ingesting either 0.2 g kg^−1^ BM NaHCO_3_ (SBC2) or 0.3 g kg^−1^ BM NaHCO_3_ (SBC3) on separate occasions, followed by a quiet rest for 180 min. Finger prick capillary blood samples were taken every 10 min, and the highest value of HCO_3_^−^ was then used as the individual time to peak. Twenty-four hours prior to each cycling TT, participants refrained from consumption of alcohol and caffeine, any strenuous activity, and maintenance of nutritional intake, which was confirmed via use of written nutrition/training diaries. Finally, participants were verbally screened to ensure avoidance of beta alanine ingestion had not occurred prior to enrolment onto the study, to account for the long washout of carnosine (Baguet et al. 2009).

### Time trial protocol, supplementation of sodium bicarbonate, and blood measures

Participants completed 2 × 4 km TTs (TT_1_ and TT_2_) interspersed with a 40 min recovery. The protocols for the TT, including the self-selected warm-up, were identical to those detailed in the previous research (Gough et al. 2017c), whereby only cadence and gear was displayed to the participant. Each TT series was preceded by supplementation of one of the SBC2, SBC3, or a taste-matched placebo (0.07 g kg^−1^ BM sodium chloride; PLA) administered in a block-randomized order. The randomisation of supplements was carried out by an individual who was not involved in the research, and performance times remained double-blind until completion of the study. Participants remained seated until their respective pre-determined time to peak HCO_3_^−^ in normoxic conditions. Once reached, participants then entered the normobaric hypoxic chamber (FiO_2_ 14.5%) for 10 min, prior to beginning the TT_1_ warm-up. Following TT_1_, participants completed a passive recovery entailing a quiet seated rest for 40 min within the hypoxic environment. Finger prick capillary blood samples for acid–base balance (pH and HCO_3_^−^), electrolytes (K^+^, Na^+^, Ca^2+^, and Cl^−^), lactate, and haemoglobin saturation with oxygen (SpO_2_) were recorded pre-exercise, at time to peak HCO_3_^−^, immediately post-exercise and at 10 min intervals during the 40 min passive recovery. These blood samples were immediately analysed using a reliable blood gas analyser (ABL800BASIC, Radiometer Medical ltd., Denmark), apart from lactate, which was analysed using a reliable and accurate lactate pro 2 analyser (Arkray, Japan) (Pyne et al. 2000; Bonaventura et al. 2015). To maintain the double-blind nature of the study, the screen on the blood gas analyser was covered, and an individual who was not part of the research stored the data until completion of the study. Calculation of the apparent SID was conducted using the formula ([K^+^] + [Na^+^] + [Ca^2+^] + [Na^+^] − [Cl^−^] − [Lac^−^]) (Lloyd 2004). Afterwards, participants completed the TT_2_ warm-up, followed by TT_2_. Additional blood samples were obtained following the warm-up in TT_2_ and immediately post TT_2_ for the same measures previously described.

### Perceptual measures

Rating of perceived exertion for overall body exertion (RPE_O_) and the legs (RPE_L_) was recorded during TT_1_ and TT_2_ every 1 km, whilst HR and SPO_2_ were recorded during both TT_1_ and TT_2_ at every 500 m split and at 10 min intervals during recovery. Gastrointestinal (GI) discomfort was recorded at 10 min intervals after ingestion of the supplement (i.e., SBC2, SBC3, or PLA) up to the individual time to peak HCO_3_^−^, and at 10 min intervals during the 40 min recovery. At individual time to peak HCO_3_^−^, participants were asked if they could determine which supplement they had ingested using a supplement belief questionnaire.

### Statistical analysis

No evidence of a violation for normality and sphericity was evident in any assessed variable, and therefore, the appropriate parametric statistical tests were employed. A paired *t* test was conducted for the following: both the time to peak and absolute change in pH and HCO_3_^−^, and both the severity and aggregated score for GI discomfort following SBC treatments. Performance data (time to TT completion and mean power) and blood parameters (change in pH and HCO_3_^−^ during TT_1_, recovery, and TT_2_) were analysed using a repeated measures ANOVA. In addition, magnitude-based inferences (MBI) with 90% confidence intervals (CI) were calculated for performance data and interpreted using an adapted method from a freely available spreadsheet (Batterham and Hopkins 2006). The thresholds to depict a benefit or harm were set as the typical error of the 4 km TT when converted from a percentage to an absolute value. This was completed by calculating the difference score in each individual, then calculating the standard deviation of the difference scores, and finally by dividing this by √2 (Swinton et al. 2018). This was used as in many cases, the 0.2 small effect size of Cohen *d* (Cohen 1988) is less than the typical error and, therefore, produces inflated positive results. Otherwise, a two-way [treatment × time] repeated measures ANOVA was conducted with a Bonferroni correction. Effect size for interactions is reported as partial eta squared (P*η*^2^) and where appropriate, between treatment Hedge’s *g* effect sizes (*g*) are reported and interpreted as per conventional thresholds (Cohen 1988). Significant effects are displayed with 95% CI where appropriate. Reproducibility of the absolute changes in pH and HCO_3_^−^ in the preliminary trial and the subsequent cycling trials was assessed using intraclass correlation coefficients (ICC). Data are reported as mean ± standard deviation (SD) and statistical significance was set at *p* < 0.05. Data were analysed using a statistical software package, SPSS (V.22, IBM Inc., Chicago, IL, USA).

## Results

### Preliminary trials to determine time to peak blood bicarbonate

Time to peak pH ranged between 30 and 100 min in SBC2 (mean 66 ± 22 min; median 60 min; CV 34%) and between 40 and 120 min in SBC3 (mean 76 ± 21 min; median 75 min; CV 27%; *p* = 0.04). The absolute change from baseline to peak pH was similar in SBC2 and SBC3 (0.08 ± 0.02 vs. 0.09 ± 0.02; *p* = 0.27). In the subsequent cycling trials, the reproducibility of the absolute change in pH was fair in SBC2 (*r* = 0.50, *p* = 0.09) and good in SBC3 (*r* = 0.60, *p* = 0.06). Time to peak HCO_3_^−^ was achieved between 30 and 110 min in SBC2 (mean 67 ± 21 min; median 60 min; CV 31%) compared to between 50 and 100 min in SBC3 (mean 77 ± 17 min; median 75 min; CV 22%; *p* = 0.20). The absolute change from baseline was greater (+ 1 mmol l^−1^) in SBC3 compared to SBC2 (7.1 ± 1.2 vs. 6.0 ± 0.9 mmol l^−1^; *p* = 0.04; *g* = 1.0). In the subsequent cycling trials, the reproducibility of the change from baseline to peak HCO_3_^−^ was good in SBC2 (*r* = 0.70, *p* = 0.04) and excellent in SBC3 (*r* = 0.77, *p* = 0.02).

### Performance

**Fig. 1:**
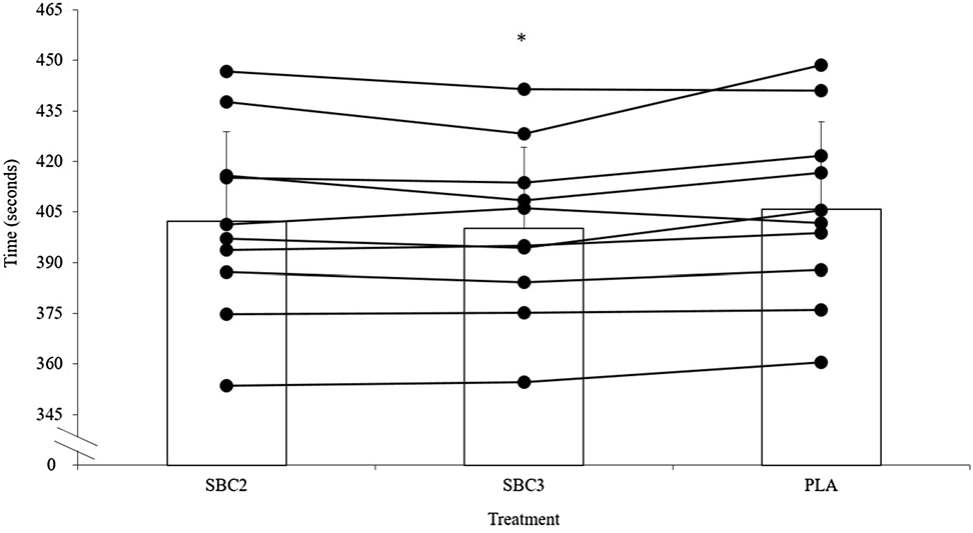
Mean (± SD) and individual (horizontal lines) time to complete time trial 1 (TT_1_) following SBC2, SBC3, and PLA. Asterisk denotes significantly improved compared to PLA (*p* < 0.05)

**Fig. 2:**
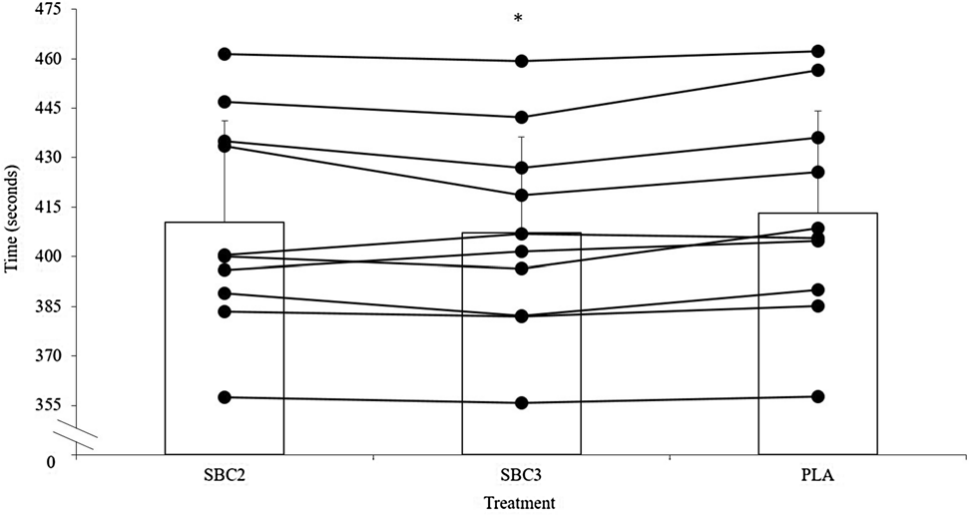
Mean (± SD) and individual (horizontal lines) time to complete time trial 2 (TT_2_) following SBC and PLA treatments. Asterisk denotes significantly improved compared to PLA (*p* < 0.05)

The decline in performance from TT_1_ to TT_2_ was similar in all treatments (SBC2 8.0 ± 6.8 vs. SBC3 7.0 ± 6.3 vs. PLA 7.3 ± 6.4 s; *p* > 0.05). In TT_1_, SBC3 improved performance compared to PLA by 1.4 ± 1.5% (400.2 ± 24.1 vs. 405.9 ± 26.0 s; *p* = 0.03; CI = 10.6, 0.8; *g* = 0.2; Fig. 1), which was determined as a very likely benefit in MBI analysis. Meanwhile, SBC2 displayed a likely benefit compared to PLA, improving performance by 0.9 ± 1.1% (402.3 ± 26.5 s; *p* = 0.14; *g* = 0.1; Fig. 1. A likely benefit was also observed in SBC3 vs. SBC2 (*p* = 0.15; *g* = 0.1). Findings were similar in TT_2_, where SBC3 again displayed the fastest completion times by 1.4 ± 1.1% compared to PLA (407.2 ± 29.2 vs. 413.2 ± 30.8 s; *p* = 0.01; CI = 10.5, 1.5; *g* = 0.2), which MBI analysis determined this as a very likely effect. Whereas, SBC2 improved performance by 0.7 ± 1.2% compared to PLA (410.3 ± 30.8 s) and this was determined as a likely benefit (*p* = 0.35; *g* = 0.1; Fig. 2). A possible benefit was determined for SBC3 compared to SBC2 for TT_2_ completion time (*p* = 0.44; *g* = 0.1).

Mean power in TT_1_ was 4.1% greater in SBC3 compared to PLA (247 ± 41 vs. 258 ± 41; *p* = 0.03; CI = 1.7, 19.6; *g* = 0.3), showing a very likely improvement. Meanwhile, SBC2 improved mean power by 2.5% compared to PLA, also revealing a very likely benefit (vs. 254 ± 43 W; *p* = 0.68; *g* = 0.2). A likely benefit was determined for SBC3 vs. SBC2 (*p* = 0.39; *g* = 0.1). Mean power in TT_2_ was improved by 3.8% in SBC3 compared to PLA (247 ± 46 vs. 237 ± 47 W; *p* = 0.005; CI = 3.0, 15.5; *g* = 0.2), and demonstrated a most likely benefit. Whereas, a likely benefit was determined in SBC2 (vs. 242 ± 47 W; *p* = 0.34; *g* = 0.1). A likely benefit was determined for SBC3 vs. SBC2 (*p* = 0.48; *g* = 0.1).

### Blood responses

**Fig. 3:**
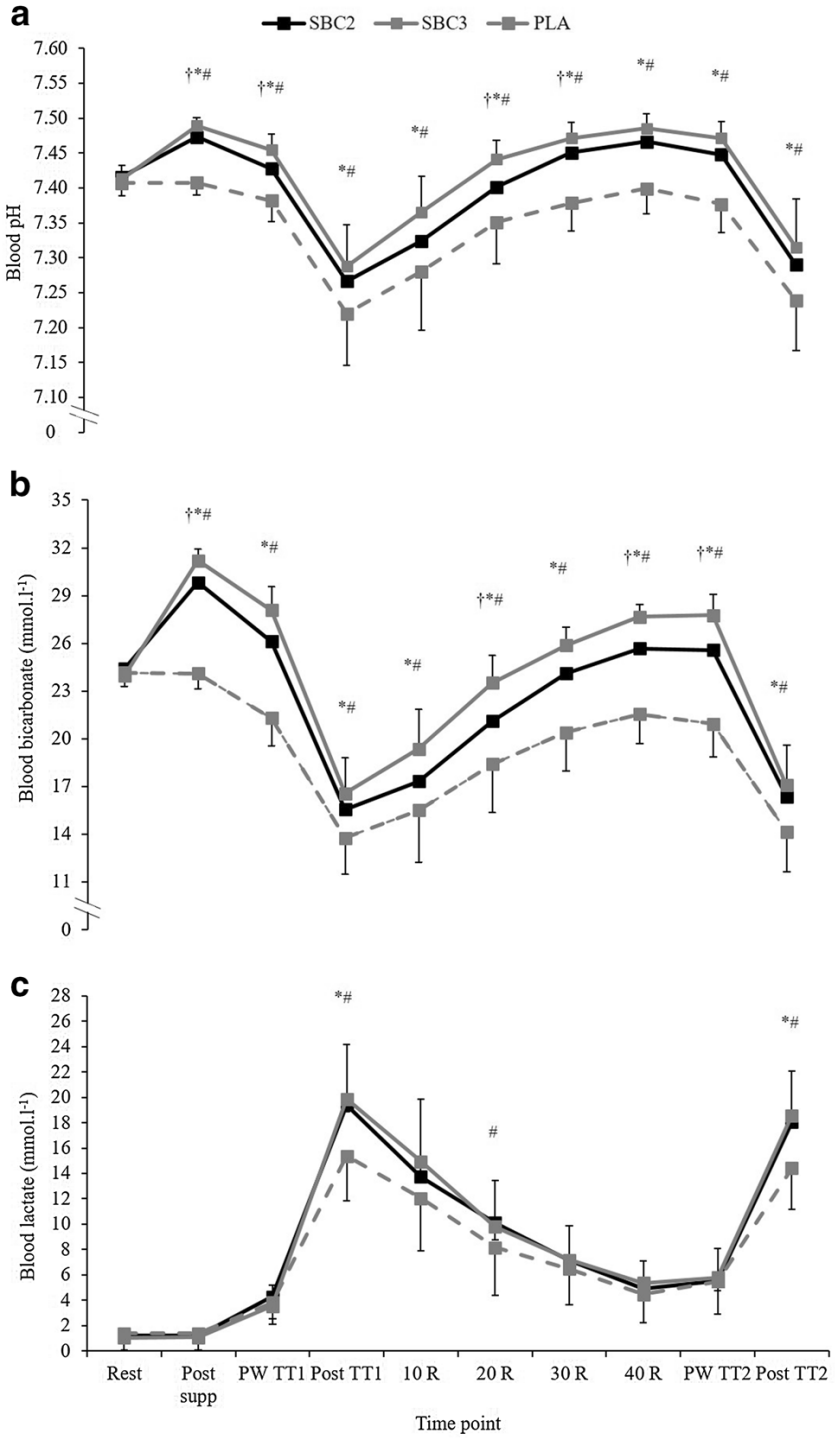
Mean (± SD) responses for blood **a** pH, **b** bicarbonate (HCO_3_^−^), and **c** lactate following NaHCO_3_ across time. SBC3 (asterisk) and SBC2 (hash symbol) significantly greater than PLA. SBC3 (dagger symbol) significantly greater than SBC2 (*p* < 0.05). *R* recovery, *PW* post warm-up

A [treatment × time] interaction was observed for HCO_3_^−^ (P*ƞ*^2^ = 0.65, *p* < 0.001), as HCO_3_^−^ was greater post-supplementation of NaHCO_3_ in SBC3 compared to both SBC2 (*p* = 0.02; CI = 0.3, 2.5, *g* = 1.5) and PLA (*p* < 0.001; CI = 6.3, 7.9; *g* = 8.4; Fig. 3). Whereas, SBC2 was greater than PLA only (*p* < 0.001; CI = 4.4, 7.1; *g* = 5.7). Post TT_1_, HCO_3_^−^ was greater in both SBC2 and SBC3 compared to PLA (both *p* < 0.001), with no differences between SBC conditions (*p* = 0.38). There was a [treatment] effect for HCO_3_^−^ change during TT_1_ (P*ƞ*^2^ = 0.69, *p* < 0.001), whereby both SBC2 and SBC3 were greater than PLA (*p* < 0.005), with a small effect size between SBC treatments (10.6 ± 3.4 vs. 11.5 ± 3.2 mmol l^−1^; *p* = 0.63; *g* = 0.26). A significant [treatment × time] interaction was observed for pH (P*ƞ*^2^ = 0.36, *p* = 0.002), as pH was greater post-supplementation and post-TT_1_ warm-up in SBC3 compared to both SBC2 and PLA (both *p* < 0.01), whilst SBC2 was greater compared to PLA (*p* < 0.001). Blood lactate was greater post-TT_1_ in both SBC treatments compared to PLA (*p* < 0.005), with no differences observed otherwise (all *p* > 0.05; Fig. 3).

**Fig. 4:**
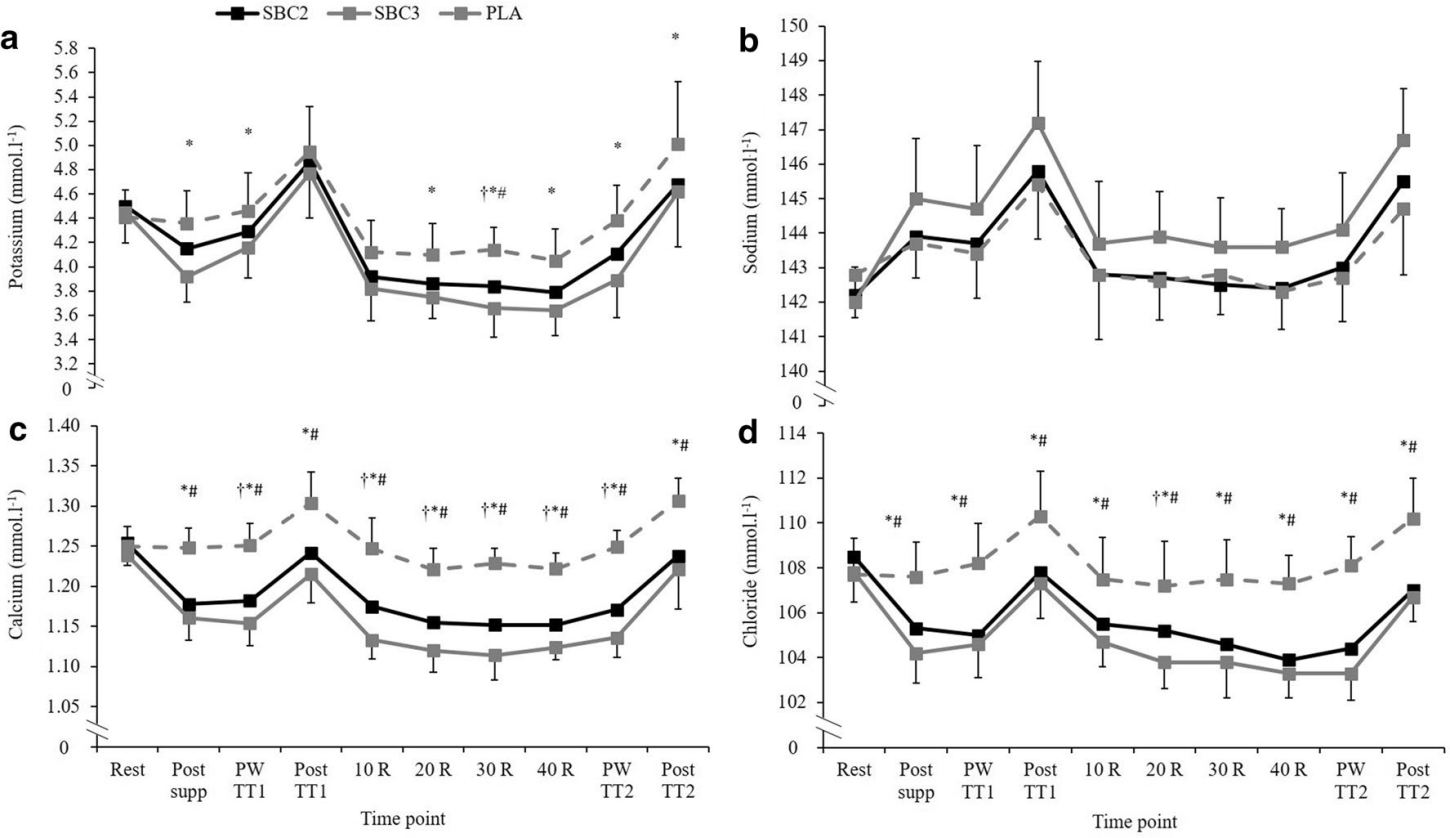
Mean (± SD) potassium (**a**), sodium (**b**), calcium (**c**), and chloride (**d**) responses over time following SBC treatments. SBC3 (asterisk) and SBC2 (hash symbol) significantly different compared to PLA. SBC3 (dagger symbol) significantly different compared to SBC2 (*p* < 0.05). *R* recovery, *PW* post warm-up

**Fig. 5:**
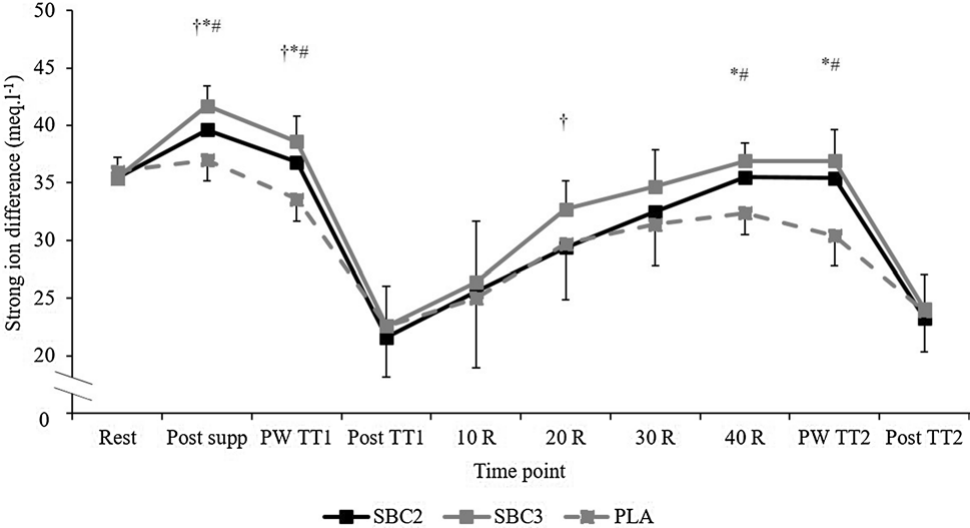
Mean (± SD) strong ion difference (SID) responses over time following SBC treatments. SBC3 (asterisk) and SBC2 (hash symbol) significantly greater than PLA. SBC3 (dagger symbol) significantly greater than SBC2 (*p* < 0.05). *R* recovery, *PW* post warm-up

Both SBC treatments elicited reductions in K^+^, Ca^2+^, and Cl^−^, and increases in Na^+^ compared to PLA (Fig. 4). The SID at post-TT_1_ warm-up and post-TT_1_ was greater in both SBC treatments compared to PLA (*p* < 0.005). However, the SID in SBC3 was greater than SBC2 post-NaHCO_3_ supplementation (*p* = 0.005; CI = 0.7, 3.5; *g* = 1.0) and post-TT_1_ warm-up (*p* = 0.049; CI = 0.01, 3.6; *g* = 0.8; Fig. 5). During recovery, HCO_3_^−^ was greater following SBC3 compared to PLA at all recovery timepoints (*p* < 0.01), however, only greater at 20 (23.5 ± 1.7 mmol l^−1^; 21.1 ± 2.7 mmol l^−1^; *p* = 0.04; CI = 0.2, 4.7; *g* = 1.0) and 40 min (27.7 ± 0.8 vs. 25.7 ± 1.3 mmol l^−1^; *p* = 0.006; CI = 0.6, 3.3; *g* = 1.8) compared to SBC2. Similarly, SBC2 was greater than PLA at all recovery timepoints (all *p* < 0.01). The absolute change in HCO_3_^−^ from post TT_1_ to 40 min recovery was significantly greater compared to PLA (5.0 ± 1.5 mmol l^−1^) in both SBC2 (10.1 ± 1.4 mmol l^−1^, *p* < 0.001; CI = 3.2, 7.1; *g* = 3.4) and SBC3 (11.1 ± 2.5 mmol l^−1^, *p* < 0.001; CI = 4.2, 8.1; *g* = 2.8), with a small effect size between SBC treatments (*p* = 0.45; *g* = 0.3). The absolute change in the SID from post-TT_1_ to 40 min recovery was only significantly greater for SBC3 compared to PLA (*p* = 0.05; CI = 0.01, 9.0; *g* = 1.2).

Post warm-up in TT_2_, HCO_3_^−^ was greater in both SBC2 and SBC3 compared to PLA (*p* < 0.001); however, SBC3 was greater than any treatment (*p* < 0.001). There was a [treatment] effect for HCO_3_^−^ change during TT_2_ (P*ƞ*^2^ = 0.71, *p* < 0.001), whereby both SBC treatments were greater than PLA (*p* < 0.01); however, SBC3 was greater compared to SBC2 (10.7 ± 2.9 vs. 9.2 ± 2.7 mmol l^−1^; *p* = 0.02; CI = 0.3, 2.6; *g* = 0.5; Fig. 3). Post-TT_2_ warm-up, and post-TT_2_, pH in both SBC2 and SBC3 were greater than PLA (*p* < 0.001), although no differences between SBC treatments were observed (*p* > 0.05). Blood lactate was greater post-TT_2_ in both SBC2 (18.0 ± 4.2 vs. 14.4 ± 3.3 mmol l^−1^; *p* = 0.05; CI = − 0.01, 7.2; *g* = 0.9) and SBC3 (18.6 ± 3.5 mmol l^−1^; *p* = 0.009; CI = 1.1, 7.2; *g* = 0.9) compared to PLA, with no difference between SBC treatments (*p* = 0.424; *g* = 1.2). Post-TT_2_ warm-up, the SID was greater for both SBC2 and SBC3 compared to PLA (*p* < 0.001); however, no difference was observed between SBC treatments (SBC2 35 ± 3 vs. SBC3 37 ± 3 meq/L; *p* = 0.20; *g* = 0.6).

### Perceptual responses

During TT_1_, NaHCO_3_ did not affect RPE_O_ (P*ƞ*^2^ = 0.24, *p* = 0.07) or RPE_L_ (P*ƞ*^2^ = 0.10, *p* = 0.38), HR (P*ƞ*^2^ = 0.07, *p* = 0.63), or SPO_2_ (P*ƞ*^2^ = 0.18, *p* = 0.16). Similarly, in TT_2_, no changes in RPE_O_ (P*ƞ*^2^ = 0.17, *p* = 0.12), RPE_L_ (P*ƞ*^2^ = 0.11, *p* = 0.35), HR (P*ƞ*^2^ = 0.07, *p* = 0.78), or SPO_2_ (P*ƞ*^2^ = 0.02, *p* = 0.83) were observed.

### Gastrointestinal (GI) discomfort

**Fig. 6:**
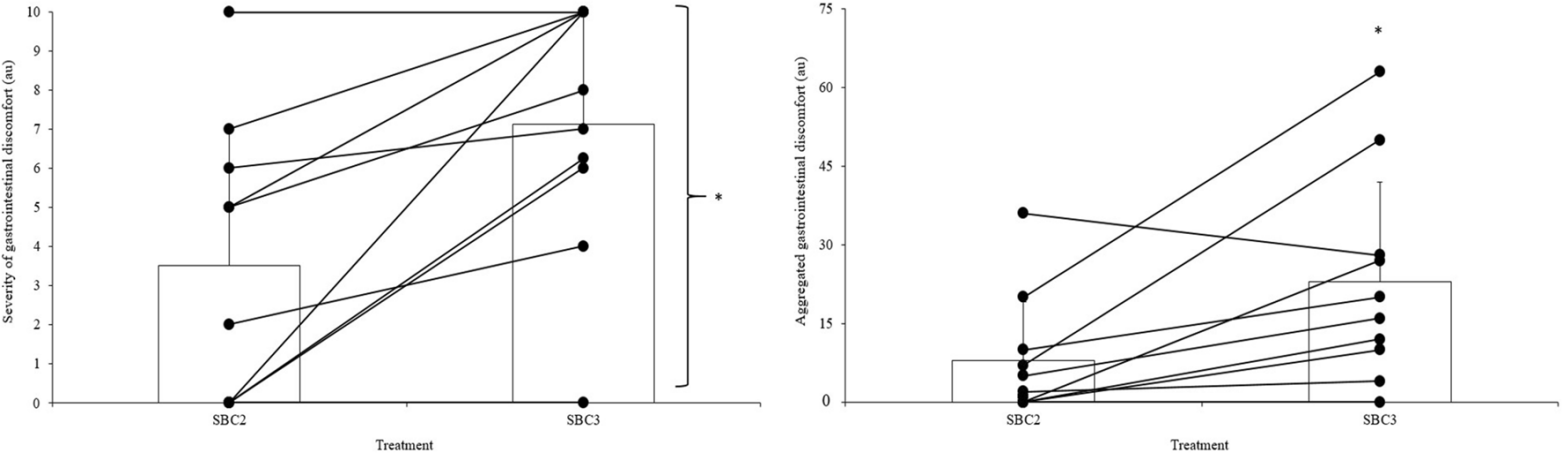
Individual aggregated score and severity of gastrointestinal (GI) responses following SBC treatments. Asterisk denotes SBC3 significantly greater than SBC2 (*p* < 0.05)

In total, 6/10 (60%) participants suffered from GI discomfort in SBC2, whereas 9/10 (90%) suffered from GI discomfort in SBC3. The most common GI discomfort symptom was belching (2/10) in SBC2, whilst in SBC3, diarrhoea, bowel urgency, and feeling of vomiting were most common (5/10). Both the aggregated GI discomfort and the severity of the most severe GI discomfort symptom suffered were greater in SBC3 compared to SBC2 (both *p* < 0.05, Fig. 6). On 3/30 (10%) occasions, the supplement was correctly identified by the participant.

## Discussion

This study investigated the effects of NaHCO_3_ ingestion on post-exercise acid–base balance recovery and repeated 4 km TT performance in moderate acute hypoxic conditions. Both SBC2 and SBC3 improved TT_1_ and TT_2_ performance compared to PLA, displaying ‘likely’ and ‘very likely’ beneficial effects in magnitude-based inferences analysis, respectively. The current study findings suggest that this occurred due to the greater magnitude of acid–base balance recovery between TT_1_ and TT_2_ or that the initial acid–base balance stress during TT_1_ was blunted by NaHCO_3_ ingestion. A greater magnitude of performance improvement was observed in SBC3, however, showing ‘likely’ and ‘possibly’ beneficial effects in TT_1_ and TT_2_ compared to SBC2, respectively. As such, SBC3 is the most optimal to improve repeated efforts of high-intensity exercise in acute moderate hypoxic conditions. One individual did display an ergolytic effect following SBC3, however, despite still gaining ergogenic effects from SBC2 vs. PLA, possibly caused by the onset of severe GI discomfort (Saunders et al. 2014). Individuals who display similar responses may, therefore, wish to select SBC2.

The current study findings suggest the enhanced exercise performance in both bouts of exercise following NaHCO_3_ ingestion occurred due to the combination of the greater alkalotic state of the acid–base balance prior to TT_1_ and the greater magnitude of acid–base balance recovery prior to TT_2_. The change in HCO_3_^−^ during TT_1_ was increased following NaHCO_3_ (SBC2 + 29%, SBC3 + 34%), whilst post TT_1_, blood lactate was also greater compared to PLA (SBC2 + 21%, SBC3 + 23%). These changes suggest an increased H^+^ buffering from intramuscular to extracellular compartments, which may lead to an increased anaerobic energy provision and glycogen utilization, as intramuscular pH is better protected (Lopes-Silva et al. 2018; Percival et al. 2015). It is argued that these indirect biomarkers of upregulated glycolytic flux in the present study instead show a reduction in lactate by inactive tissue however, as the samples in the present study do provide data on use/uptake (Granier et al. 1996). Likewise, it is strongly argued that acidosis does not inhibit contractile machinery and that alkalization may cause instability of the buffering systems, and thus, the benefit to the ATP-generating process is either negligible, or actually harmful (Korzeniewski and Zoladz 2002; Sahlin et al. 1998). A recent study by Lopes-Silva et al. (2018), nonetheless, reported similar post-exercise HCO_3_^−^ and lactate responses to the current study following NaHCO_3_ ingestion, yet also reported both a 34% greater estimated glycolytic energy contribution to exercise and an improved performance. It is alternately conceivable that the increases in HCO_3_^−^ and CO_2_ following NaHCO_3_ ingestion are part of a causal sequence of the primary mechanism of performance, by accelerating *V*O_2_ kinetics at the onset of high-power outputs (Zoladz et al. 2005). Unfortunately, *V*O_2_ was not measured during the TTs in the study, so this cannot be concluded and thus warrants further research. The current study findings, nonetheless, further support past research (Fitts 2016) that acid–base balance disturbances are an important determinant of fatigue during high-intensity exercise.

The current study adds that NaHCO_3_ ingestion increased the SID prior to each TT bout, which may explain the improved performance. The SID has been suggested to be an important component of action potentials by increasing muscle excitability (Allen et al. 2008). Notable changes NaHCO_3_ ingestion elicits include a lowering of extracellular K^+^ and Cl^−^, whilst increasing Na^+^, all of which have collectively been identified to be important for muscle contraction during high-intensity exercise previously (Cairns and Lindinger 2008). Here, the current study findings expand upon those of Sostaric et al. (2006) that reported an improvement in finger flexion to exhaustion combined with an increased SID following NaHCO_3_ ingestion, by showing a similar effect but during dynamic whole-body exercise. These findings also provide an alternative mechanism to the pH and HCO_3_^−^-mediated mechanisms often criticised in the literature (Westerblad 2016). It is worth noting that the highlighted changes in the SID may also have further reaching benefits to health-related outcomes. Mild metabolic acidosis has been linked with multiple health-related outcomes including protein metabolism, by decreasing synthesis through increases of non-enzymatic proteolysis (Wiederkehr and Krapf 2001). Likewise, regular consumption of alkaline rich supplements has been shown to improve mineral balance and rate of bone formation (Sebastian et al. 1994). Therefore, more research is required investigating the use of chronic NaHCO_3_ ingestion in sporting populations that could also be prone to such issues, particularly endurance runners and adolescent athletes (Tenforde et al. 2017; Scofield and Hecht 2012).

Both pH and HCO_3_^−^ following SBC treatments were increased prior to TT_2_, such that the absolute change in HCO_3_^−^ from TT_1_ to 40 min recovery was over twofold greater compared to PLA (SBC2 + 51%, SBC3 + 55%), whilst the change in the SID was also more superior (SBC2 + 29%, SBC3 + 31%). This suggests a greater amount of H^+^ buffering occurred during this time, which subsequently facilitated a more substantial recovery of acid–base balance compared to PLA, in support of previous research (Pruscino et al. 2008; Gough et al. 2017a). Alternatively, the improvement in TT_2_ may have been due to the stress on acid–base balance being blunted by NaHCO_3_ during TT_1_, as pH, HCO_3_^−^, and the SID were all greater immediately post TT_1_ compared to PLA. Based on data from Gough et al. (2017a), however, it is more likely that the enhancement of the acid–base balance state between TT_1_ and TT_2_ explains the improvement in the current study, as the authors reported NaHCO_3_ ingestion improved a subsequent exercise even when supplementation was after an initial bout of exercise. Moreover, SBC3 elicited a significantly greater magnitude of acid–base balance recovery prior to TT_2_, and change in HCO_3_^−^ during TT_2_ compared to SBC2. Correspondingly, SBC3 improved performance within half of the sample compared to SBC2, compared to only two displaying greater improvements in SBC2 vs. SBC3 when using the 3.1 s TE of the test. Combined, these greater increases in acid–base balance blood analytes may explain the greater magnitude of improvement in TT_2_ produced by SBC3.

At 40 min recovery pH, HCO_3_^−^ and the SID were still rising following NaHCO_3_ ingestion, such that pH at 40 min recovery was 7.49 ± 0.02 in SBC3, which was identical to the increase prior to TT_1_ following the same dose (7.49 ± 0.01). This is in agreement with the previous research in normoxia showing similar increases at the end of a recovery period typically seen with pre-exercise NaHCO_3_ ingestion (Callaghan et al. 2017; Pruscino et al. 2008). Equally, in the current study, and others (Callaghan et al. 2017; Pruscino et al. 2008), acid–base balance status was still significantly rising to a more alkalotic state. It is plausible to suggest, therefore, if a longer period of recovery was employed, a more pronounced performance effect may have been observed compared to PLA. Moreover, this also suggests that re-dosing NaHCO_3_ following an initial fatiguing bout is not required, as acid–base balance increased well above baseline, despite no re-dosing of NaHCO_3_. These findings may be of importance to individuals who suffer from GI discomfort, as no instances were reported during recovery in the current study. Future research should, therefore, investigate the performance responses on repeated exercise following NaHCO_3_ ingestion with a longer period of recovery.

One participant presented an ergolytic effect in both TT_1_ and TT_2_ after ingestion of SBC3. This was likely due to the occurrence of severe GI discomfort (diarrhoea = 10; aggregate score = 63), as this participant still improved their performance in SBC2. These findings support the previous research, whereby ergolytic effects were observed in participants who suffered from severe GI discomfort following 0.3 g kg^−1^ BM NaHCO_3_ (Saunders et al. 2014; Froio de Araujo Dias et al. 2015). It is therefore important to monitor the GI discomfort responses following NaHCO_3_ ingestion on an individual basis, as those who display severe symptoms following SBC3 may instead benefit from ingesting SBC2. The use of this smaller dose, however, will be dependent on an improvement in performance still being observed compared to a placebo.

## Conclusion

This study investigated the effects of NaHCO_3_ ingestion on repeated 4 km TT performance and acid–base balance recovery in acute moderate hypoxic conditions. Both amounts of NaHCO_3_ employed in this study ensured recovery of acid–base balance back to baseline or above within 20–40 min, whereas this was not achieved for PLA. For the first time, blunting of the acid–base balance stress during the initial bout of exercise, or a greater magnitude of acid–base balance recovery, has translated into improved subsequent high-intensity exercise performance following NaHCO_3_ ingestion in acute hypoxic conditions. The performance improvement was greater in SBC3, which is likely due to the greater alkalotic status of acid–base balance both prior to TT_1_, and during recovery compared to SBC2 and PLA. The onset of GI discomfort was an issue with SBC3, however, and one participant displayed an ergolytic effect on performance following this dose. Individuals should, therefore, employ SBC3 to improve performance in acute hypoxic conditions, only if severe GI discomfort does not occur.

## Abbreviations

Ca^2+^: Calcium
Cl^−^: Chloride
CI: Confidence intervals
FiO_2_: Fraction of inspired oxygen
GI: Gastrointestinal
HCO_3_^−^: Bicarbonate anion concentrations
H^+^: Hydrogen cation
K^+^: Potassium
SID: Strong ion difference
*V*O_2max_: Maximal rate of oxygen uptake
Na^+^: Sodium
O_2_: Oxygen
SpO_2_: Haemoglobin saturation of oxygen
NaHCO_3_: Sodium bicarbonate
NaCl: Sodium chloride
TT: Time trial
